# Defining Healthy Weight Loss and Target Weight in the Era of Highly Effective Treatment of Patients With Obesity

**DOI:** 10.1002/jcsm.70334

**Published:** 2026-07-15

**Authors:** Anja Bosy‐Westphal, Janna Enderle, Kristina Norman, Manfred J. Müller

**Affiliations:** ^1^ Department of Human Nutrition, Institute of Human Nutrition and Food Science Kiel University Kiel Germany; ^2^ German Institute of Nutritional Science, University of Potsdam, Department of Nutrition and Gerontology, German Institute of Human Nutrition Potsdam‐Rehbrücke Nuthetal Germany; ^3^ Department of Geriatrics and Medical Gerontology, Charité‐Universitätsmedizin Berlin, Corporate Member of Freie Universität Berlin and Humboldt‐Universität zu Berlin Berlin Germany

**Keywords:** anti‐obesity medication, fat‐free mass, fat mass, partitioning, skeletal muscle mass, weight loss

## Abstract

**Background:**

Highly effective anti‐obesity medications (AOMs) induce substantial weight loss and have renewed debate on appropriate treatment targets. Weight loss comprises reductions in fat mass (FM) and fat‐free mass (FFM), with skeletal muscle (SM) as the major component of FFM. Clinical trials report that 20%–40% of total weight loss is attributable to FFM, raising concerns about excessive SM loss and sarcopenia risk.

**Methods:**

This narrative review integrates physiological principles and quantitative data from clinical trials to derive a rationale for redefining healthy weight loss targets based on body composition. Determinants of energy partitioning (baseline adiposity, age, diet and physical activity) are combined with predictive and reference‐based approaches. Skeletal muscle index (SMI) was estimated from FFM, body weight, sex and age (*R* ^2^ = 0.87, SEE = 0.635 kg/m^2^) and evaluated using SMI standard deviation scores (SDS).

**Results:**

SM accounts for 92%–97% of FFM loss during weight reduction. In incretin‐based trials, FM decreased by −18.7% to −33.9% and FFM by −6.8% to −10.9%, corresponding to 33%–38% of total weight loss. Model‐based estimates predict lower FFM reductions (~6.8%–6.9%), indicating that observed FFM losses exceed physiological expectations under conditions of large energy deficits. Across studies, diet‐ or AOM‐induced weight loss without structured exercise is associated with higher proportions of FFM loss than expected, whereas interventions including exercise show more favourable partitioning. An SMI‐SDS threshold of −0.67 (25th percentile) identifies clinically relevant SM depletion.

**Conclusions:**

Disproportionate SM loss is common with considerable weight reduction and may exceed physiologically expected levels. An SMI‐based framework enables definition of individualized, safe treatment targets and may help prevent sarcopenia during obesity treatment.

**Abbreviations**
ADP: Air displacement plethysmography
ALST: appendicular lean soft tissue
AOM: Anti‐Obesity Medication
AT: adipose tissue
BIA: bioelectrical impedance analysis
BMI: body mass index
DXA: dual‐X‐ray absorptiometry
FFM: fat‐free mass
FFMI: fat‐free mass index
FFMV: fat‐tissue free muscle volume
FM: fat mass
FMI: fat mass index
IRA: incretin receptor agonists
L3: lumbar vertebrae 3
MRI: magnetic resonance imaging
SM: skeletal muscle mass
SMI: skeletal muscle index

## 1 Introduction

The introduction of incretin receptor agonists has significantly advanced the management of obesity and its associated comorbidities [1]. The substantial weight reductions achieved with these agents have reignited discussions on how treatment success in obesity should be assessed [2, 3, 4, 5, 6]. However, similar to body mass index (BMI), weight loss alone does not constitute an adequate therapeutic target [7]. This is underscored by the observation that obesity‐related complications may occur even at BMI values below 25 kg/m^2^, driven by adipose tissue dysfunction [8]. Evidence suggests that each individual possesses a personal fat threshold, beyond which the accumulation of intrahepatic and intrapancreatic fat impairs insulin secretion, exacerbates insulin resistance and thereby increases the risk of type 2 diabetes (T2D) [9, 10, 11]. Healthy weight loss should therefore prioritise reducing adipocyte overload and ectopic lipid storage rather than simply lowering total fat mass (FM) [12].

The so‐called obesity paradox—increased survival at higher BMI—has been attributed to greater fat‐free mass (FFM) rather than excess FM [7]. Skeletal muscle (SM), the largest component of FFM, is central to protein turnover and metabolic health. It secretes exercise‐associated exerkines that promote cardiovascular, metabolic, immune and neurological function [13, 14]; supports immune defence [15]; and preserves independence through strength [16]. Metabolically, SM accounts for ~50%–70% of glucose disposal after carbohydrate intake [17], ~22% of resting energy expenditure [18, 19] and significantly contributes to whole body fat oxidation [20, 21, 22]. Preserving SM during weight loss is therefore critical to maintaining energy expenditure and reducing the risk of weight regain that is commonly observed after lifestyle interventions [23], bariatric surgery [24] or discontinuation of pharmacotherapy [25].

In contrast to lifestyle‐based interventions, anti‐obesity medications (AOM) may predispose to disproportionate FFM loss through reduced appetite for protein‐rich foods [26], gastrointestinal side effects that suppress food intake [27] or glucagon‐mediated protein catabolism [28]. Glucagon may lower circulating amino acids, reducing protein synthesis [29]. Conversely, GLP‐1 receptor agonists increase glucose‐stimulated insulin secretion [30], and weight loss itself improves insulin sensitivity [31], potentially mitigating muscle catabolism.

Concerns over excessive FFM loss are not new: Bariatric surgery has long raised such issues [32, 33]. AOMs, however, are accessible to broader patient groups and are increasingly used—even repeatedly or as lifestyle drugs in people without clear medical indications [34]. While some authors highlight risks of excessive SM loss [5, 35], others have not found supporting evidence [2, 36, 37]. To date, weight loss‐induced body composition changes under AOM have been described only quantitatively (e.g., the proportion of FFM lost relative to total weight loss), without normalization for the physiological determinants of FM and FFM changes. Current data are therefore insufficient to fully evaluate AOM‐induced shifts in body composition and their health consequences.

The primary aim of this review is to propose practical guidelines for evaluating weight loss that can be applied from individual patient care to group‐level analyses. We **first** outline the physiological determinants of body composition change and then introduce two complementary adjustment strategies—dynamic and static models. The **second** aim is to define criteria for a healthy weight loss target based on body composition, a question that has become urgent with the advent of highly effective AOMs.

### 1.1 Determinants of Normal Energy Partitioning With Weight Loss

Body weight and composition prior to weight loss, along with factors such as age, sex, physical activity, dietary patterns and comorbidities like T2D, are known determinants of body composition changes during weight loss. These variables can be accounted for or controlled by matching conditions within the control group. However, when interpreting individual patient data, careful consideration of these influencing factors remains important.

#### 1.1.1 Impact of Body Composition Before Weight Loss

Baseline body composition is the strongest predictor of the composition of weight loss. This relationship can be evaluated using a two‐compartment model (distinguishing FM and FFM) based on established methods of body composition assessment, such as hydrodensitometry, air displacement plethysmography (ADP), dual‐energy X‐ray absorptiometry (DXA) and bioelectrical impedance analysis (BIA) [38]. Gilbert Forbes was the first to describe the FFM fraction of body weight change (ΔFFM/ΔBW) as a function of initial body fat percentage (%FM), highlighting the predictive role of baseline adiposity [39, 40, 41].

#### 1.1.2 Impact of Baseline Adiposity

The mechanisms underlying the Forbes partitioning rule are closely linked to metabolic adaptations in substrate oxidation during caloric and carbohydrate restriction or starvation [42]. Due to the limited capacity for glycogen storage and the essential functional role of body protein, both carbohydrate and protein balances are tightly regulated in response to changes in energy and protein intake. In contrast, fat oxidation is not acutely regulated by fat intake. Thus, energy surpluses or deficits are primarily buffered through changes in fat stores [43, 44]. The composition of oxidized substrates is therefore influenced by the size and availability of endogenous fuel stores [43].

During the first week of caloric restriction, an initial, transient increase in FFM loss is typically observed, representing losses in glycogen, water, minerals, electrolytes and protein [45]. However, during prolonged caloric restriction or starvation, SM protein is increasingly spared—reflected in a daily protein loss of only ~35–40 g—due to metabolic adaptations including enhanced lipid oxidation, ketogenesis and the use of glycerol for hepatic gluconeogenesis. This spares alanine as a gluconeogenic precursor within the glucose‐alanine cycle [46], ultimately promoting survival [47]. Forbes and Drenick (1979) [48] quantified nitrogen depletion kinetics, reporting half‐lives of 2.4 days (early phase) and 116 days (late phase) in individuals not affected by obesity. In individuals with obesity, these values extended to 10.6 and 433 days, respectively, highlighting the protein‐sparing effect of larger fat stores during sustained energy deficits [45].

#### 1.1.3 Impact of Diet and Exercise

Forbes already described that the fraction of weight lost as FFM during caloric restriction is also a function of the magnitude of the energy deficit, with a large deficit resulting in a slightly greater loss of lean mass. Therefore, the original Forbes equation has been extended to account for the weaker effect of the magnitude and direction of body weight changes to improve prediction of major body composition changes, such as in patients following bariatric surgery [49]. This is important because modern AOM can cause a drastic drop in energy intake by suppressing appetite or causing nausea [27]. Negative energy balance increases oxidation of amino acids at the expense of impaired maintenance of FFM [50]. Whole‐body protein breakdown and synthesis are both reduced with increasing duration of an energy deficit [51]. However, with prolonged energy deficit, the rate of muscle protein synthesis increases again [52, 53], suggesting that higher muscle protein degradation compared to protein synthesis may be the major cause of the loss of muscle mass during prolonged weight loss.

A high protein intake protects against the loss of FFM during weight loss [54, 55] and facilitates a gain in lean mass with weight gain [56]. It was shown that ∼22 g protein per meal (1.2 g protein/kg body weight/d) combined with resistance exercise prevented a loss in lean mass in overweight men despite a substantial energy deficit of 40%, whereas ∼49 g protein/meal (2.4 g protein/kg body weight/d) even resulted in a gain in lean mass (+1.2 ± 1.0 kg) and a greater loss in FM over 4 weeks [57]. A gain in FFM with diet‐ and exercise‐induced weight loss (∼20% energy deficit) and a protein intake of 1.33 g/kg body weight/d was also observed during a longer term intervention in premenopausal women with overweight or obesity [58]. Reduced postabsorptive muscle protein synthesis after a 5‐day energy deficit was shown to be restored by resistance training and further increased above resting energy balance with protein intake in a dose‐dependent manner with higher doses of protein > 0.25 g × kg‐1 per meal [59]. Due to ‘anabolic resistance’ to protein intake and exercise, older adults likely require larger amounts of protein for preservation of FFM during weight loss (for review see [60]). In addition, a high protein intake is also recommended with AOM despite a lack of clinical evidence [61].

Contrary to the prevailing notion that a greater carbohydrate content in the diet might hinder fat loss due to elevated insulin levels, which might impede lipolysis and consequently impair fat oxidation, a strictly controlled study by Hall et al. (2015) [62] in adults with obesity observed a slightly higher loss in FM with a high‐carbohydrate weight loss diet (71% carbohydrate, 8% fat, −30% energy deficit) that prevented the energy deficit‐induced drop in daylong insulin levels when compared with a high‐fat diet (29% CHO, 50% fat). This discrepancy can be attributed to a tendency towards a more pronounced negative energy balance during the initial phase of the high‐carbohydrate diet in comparison to the high‐fat diet. Calorie for calorie, a high‐carbohydrate weight loss diet, therefore led to a slightly greater loss in FM than a high‐fat diet, despite the fact that only the high‐fat diet led to decreased insulin secretion.

#### 1.1.4 Impact of Ageing

Age‐related loss of SM is a multifactorial process driven by insulin resistance, chronic low‐grade inflammation, hormonal alterations and lifestyle changes, with additional contributions from neuromuscular degeneration, extracellular matrix remodelling and impaired vascular perfusion [63, 64]. SM mass peaks in early adulthood, stabilizes during midlife and declines by ~1%–2% per year after age 40 [65, 66]. This reduction is often masked by parallel gains in FM, but muscle quality deteriorates as adipose tissue infiltrates contractile tissue, giving rise to functional impairments characteristic of sarcopenia.

Importantly, SM loss is not uniform across the body. Atrophy rates vary by location and function, with recent data showing more than a fivefold difference: The soleus shows only minor loss (−6%; −0.13%/year), whereas the rectus femoris is highly susceptible (−33%; −0.66%/year) [67, 68]. These findings underscore the heterogeneity of sarcopenia and the need for regional muscle mass assessment and interventions.

#### 1.1.5 Impact of Diseases

Metabolic adaptations associated with weight loss are markedly altered in catabolic disease states such as chronic heart or kidney failure, severe infections and cancer cachexia [69]. In these conditions, energy partitioning shifts in response to reduced energy intake as well as inflammatory and metabolic signals like insulin resistance, which reorient homeostatic mechanisms towards supporting immune function. This redistribution of metabolic resources leads to a disproportionate breakdown of body protein [70].

Insulin resistance and chronic low‐grade inflammation also contribute to impaired SM maintenance in individuals with T2D [71]. Longitudinal data show that adults with T2D experience a significantly greater loss of appendicular lean soft tissue (ALST) over time compared to nondiabetic controls [72]. Importantly, even in nondiabetic individuals, insulin resistance has been linked to greater lean mass loss during weight reduction [73, 74].

While measures of insulin resistance either reflect increased hepatic gluconeogenesis (i.e., an increased HOMA‐IR) or decreased insulin‐mediated glucose uptake in SM, emerging evidence suggests that insulin resistance attenuates insulin‐mediated suppression of proteolysis and stimulation of protein synthesis, even in the presence of adequate protein availability [75]. However, the aetiology of muscle loss and fibre type shifts in people with T2D is likely multifactorial, involving malnutrition, hormonal dysregulation (affecting, e.g., insulin, cortisol, prostaglandins, IGF‐1 and growth hormone), chronic inflammation and oxidative stress [76].

### 1.2 How to Evaluate Weight Loss‐Induced Changes in Body Composition: Dynamic vs. Static Approach

Changes in body composition during weight loss can be assessed using two principal approaches. The first can be termed ‘dynamic approach’ and uses preweight loss parameters to predict expected changes in FFM and FM. This can be modelled using established equations such as the Forbes model [40, 41], which estimates the FFM retention based on initial FM. For example, an individual with a BMI of 30 kg/m^2^ and a high baseline FM is predicted to retain more FFM than someone with the same BMI but lower FM, given the same degree of weight loss. The Forbes relationship is originally derived from female datasets. While the underlying principle appears to apply to both sexes, the original parameterization may not fully capture sex‐specific differences in body composition. In addition, the Forbes relationship is based on a differential formulation that is strictly valid only for infinitesimal changes in body weight and body composition [49].

A more comprehensive *dynamic* method is the mathematical model developed by Hall et al. (2011) [77]. This model accounts for baseline body composition (measured or estimated based on sex, age and BMI), the magnitude and duration of energy surplus or deficit (energy intake vs. energy expenditure) and optionally, carbohydrate intake. It enables retrospective evaluation of weight loss associated changes in body composition, especially useful when the energy deficit cannot be precisely quantified on an individual basis, as is the case with the use of AOMs. In practice, Hall's model predicts postintervention body composition by using parameters such as sex, age, height, physical activity level, initial body weight and observed postintervention weight (‘goal weight’), along with the duration of the intervention using the online available body weight planner (https://www.niddk.nih.gov/health‐information/weight‐management/body‐weight‐planner). This provides a valuable tool for estimating expected changes in FFM and FM in response to pharmacological or dietary weight loss interventions. Given that rapid and pronounced weight reduction induced by AOMs predisposes individuals to disproportionate losses of SM, early identification of patients at risk of sarcopenia is essential. Therefore, a baseline body composition assessment in clinical and outpatient settings is a prerequisite for individualized treatment strategies, such as gradual dose escalation combined with protein supplementation and exercise.

A ‘static approach’ of body composition assessment compares individual pre‐ (or post‐) weight loss body composition data with values derived from cross‐sectional studies of a healthy reference population. The ideal reference population should be applicable to the age‐ and BMI‐range, as well as ethnicity/race of the investigated population. The resulting *z*‐scores or standard deviation scores (SDS) are contingent upon the normal distribution of the data, the absence of residual associations and constant variance of the normalized measurements throughout the entire sample (absence of heteroscedasticity, logarithmic transformation of the dependent variables or weighted regression models). The individual *z*‐score or SDS‐score for FFM or SM (or normalized for height^2^ as FFMI and skeletal muscle index (SMI) in kg/m^2^, respectively) would reveal a too low SM at a negative *z*‐score or SDS‐score < −0.67 (< 25th percentile) obesity, or a muscular phenotype at a positive *z*‐score or SDS‐score > 0.67 (> 75th percentile).

Table 1 provides an overview of the methods for body composition assessment: (i) ‘static’ or ‘dynamic’ approaches, (ii) underlying methods of body composition analysis (i.e., two‐compartment methods of FM and FFM or regional measures of SM by magnetic resonance imaging, MRI) and (iii) the determinants of changes in body composition that are considered.

**TABLE 1:** Different methods for assessing changes in body composition during weight loss.

Dynamic methods	Characteristics	Body composition method, determinants, population
Forbes 2000 [41]	$\frac{\text{dFFM}}{\mathrm{dBW}}=\frac{10\cdot 4}{10\cdot 4+\mathrm{FM}}$	Considers only cross‐sectional data of females for the empirical equation, relies on a two‐compartment model
Hall 2007 [49]	$\frac{\Delta \mathrm{FFM}}{\Delta \mathrm{BW}}=1+\frac{{\mathrm{FM}}_{i}}{\Delta \mathrm{BW}}-\frac{10.4}{\Delta \mathrm{BW}}W\left\{\frac{1}{10.4}\exp\left(\frac{\Delta \mathrm{BW}}{10.4}\right){\mathrm{FM}}_{i}\exp\left(\frac{{\mathrm{FM}}_{i}}{10.4}\right)\right\}$ with BW, body weight; W, Lambert W function; FMi, initial values for FM	Extended Forbes's equation to account for the magnitude and direction of macroscopic body weight changes, relies on a two‐compartment model
Hall et al. 2011 [77]	Mathematical model for predicting the dynamics of change in weight and body composition in adults based on simulated adaptations in energy expenditure and information on changes in diet and physical activity (including web‐based simulator)	Dynamic simulation model of human metabolism that predicts the time course of body composition change (FM, FFM) at both the individual and population levels
Static methods		
Linge et al. 2020 [78]	Sex‐and‐BMI‐matched fat‐tissue free muscle volume (FFMV) quantified through MRI ➔ FFMV/height^2^ *z*‐score Number of standard deviations from the mean of a matched group (with the same sex, age and BMI)	UK Biobank resource (9615 adults, 62.6 ± 7.5 years, BMI 26.6 ± 4.4 kg/m^2^). FFMV at the thighs (MRI DIXON) was defined as the sum of all voxels with fat fraction < 50% (insensitive to erroneous segmentation in boundaries between muscle and intermuscular adipose tissue)
Heymsfield et al. 2024 [79]	Males SM = −13 + 0.38 × weight +0.10 × height +0.11 × age − 0.003 × age × weight Females SM = −7.14 + 0.14 × weight +0.12 × height—0.04 × age With SM in kg; weight in kg; height in cm; age in years.	897 healthy adults (18–88 years, BMI between 26.5 ± 3.3 in males and 25.0 ± 4.1 kg/m^2^ in females). Whole body SM volume determined by MRI, measured volumes were transformed into SM mass assuming a density of 1.04 kg/L
Heymsfield et al. 2022 [80]	Males ALST = 0.202 × BW + 0.127 × height − 0.063 × age − 10.6 Females ALST = 0.162 × BW + 0.093 × height − 0.035 × age − 7.9 With ALST (appendicular lean soft tissue) in kg; weight in kg; height in cm; age in years	8623 non‐Hispanic White adults from NHANES (18–85 years, BMI 28.1 ± 5.7 in males and 27.8 ± 6.9 kg/m^2^ in females). ALST measured by dual X‐ray absorptiometry (DXA)
Enderle et al. 2023 [81]	Males FFMI: −0.0151 age + 0.000013 age^2^ + 0.3231 BMI + 11.9 (SEE 1.04) SMI: 0.0399 age − 0.000668 age^2^ + 0.1740 BMI + 4.79 (SEE 0.55) Females FFMI: −0.0559 age + 0.000376 age^2^ + 0.2534 BMI + 11.47 (SEE 0.90) SMI: 0.0274 age − 0.000559 age^2^ + 0.1391 BMI + 3.89 (SEE 0.52) With FFMI and SMI in kg/m^2^; BMI in kg/m^2^; age, in years SDS < 0.67 ➔ measured value < 25th percentile	1958 healthy adults, 18–97 years, BMI 17.1–45.6 kg/m^2^, bioelectrical impedance analysis for prediction of FFMI (using four‐compartment model as a reference) or SMI (using whole body MRI as a reference) $\mathrm{SDS}=\frac{\text{result}-\text{predicted value}}{\mathrm{SEE}}$

Abbreviations: ALST, appendicular lean soft tissue in kg; BMI, body mass index in kg/m^2^; BW, body weight in kg; DXA, dual X‐ray absorptiometry; FFM, fat‐free mass in kg; FFMI, fat‐free mass index in kg/m^2^; FFMV, fat‐tissue free muscle volume; FM, fat mass in kg; MRI, magnetic resonance imaging; SDS, standard deviation score; SEE, standard error of estimate; SM, skeletal muscle; SMI, skeletal muscle in kg/m^2^.

### 1.3 Applications of the Dynamic and Static Approach in AOM Studies

When applying the mathematical model by Hall et al. (2011) [77] to evaluate body composition changes reported in the Tirzepatide trial [36], the predicted mean FM reductions were −27% in women and −33% in men, with corresponding decreases in FFM of −6.8% and −6.9%, respectively. These model‐derived estimates align closely with the observed −33.9% reduction in FM. However, the reported mean FFM loss of −10.9% exceeded the model predictions, although sex‐specific FFM data were not provided.

Muscle volume *z*‐scores, benchmarked against the UK Biobank reference population [78], were comparable between liraglutide and Tirzepatide at the 5 mg dose, reflecting similar magnitudes of weight loss [4]. Notably, at the highest Tirzepatide dose (15 mg), the mean muscle volume *z*‐score declined to −0.30 ± 0.47, significantly lower than predicted. Furthermore, all treatment groups (Tirzepatide 5, 10 and 15 mg and liraglutide) showed significant within‐group reductions in muscle volume *z*‐scores from baseline (*p* < 0.05). Given that participants in the SURPASS‐3 trial were individuals with T2D, the observed lower baseline muscle volume *z*‐score compared to healthy, age‐, sex‐ and BMI‐matched UK Biobank controls is expected. The disproportionately greater SM loss observed at the highest Tirzepatide dose is likely attributable, at least in part, to the larger energy deficit associated with greater weight loss. In this study, SM was evaluated using muscle volume *z*‐scores adjusted for age, sex and BMI. By design, this normalization accounts for the expected relationship between body size and SM. However, because these *z*‐scores are derived from cross‐sectional reference populations with stable body weight, individuals undergoing substantial and rapid weight loss may exhibit lower SM than expected for their BMI leading to a reduced *z*‐score. Several mechanisms may contribute to such disproportionate SM loss. Large energy deficits are known to suppress muscle protein synthesis and may increase muscle protein breakdown [50]. In addition, substantial reductions in body weight decrease the mechanical load on SM, which can further reduce anabolic signalling. Reduced protein intake [26] and fatigue‐related decreases in spontaneous physical activity during periods of rapid weight loss may also contribute to a greater relative loss of SM.

### 1.4 How to Normalize FFM or SM for Body Size and Weight Status?

FFM and SM are often normalized to body size by expressing them as a percentage of body weight (%FFM or %SM). However, this approach has several limitations. Normalizing FFM by body weight is inappropriate because two individuals with identical %FFM but different heights can still differ significantly in absolute body composition, with the taller individual exhibiting a lower relative muscularity, despite having the same %FFM [82]. Moreover, expressing FM as a percentage of body weight shifts the focus towards FM, rendering independent interpretation of FFM difficult. A high %FM inherently implies a low %FFM and vice versa, which may lead to misleading conclusions. For instance, elevated %FM may be wrongly interpreted as the primary risk factor, while in reality, reduced %SM may be the major health issue [83]. In addition, %FM changes slowly with variations in absolute FM and tends to plateau at around 60% body fat with further weight gain, limiting its sensitivity.

**FIGURE 1:**
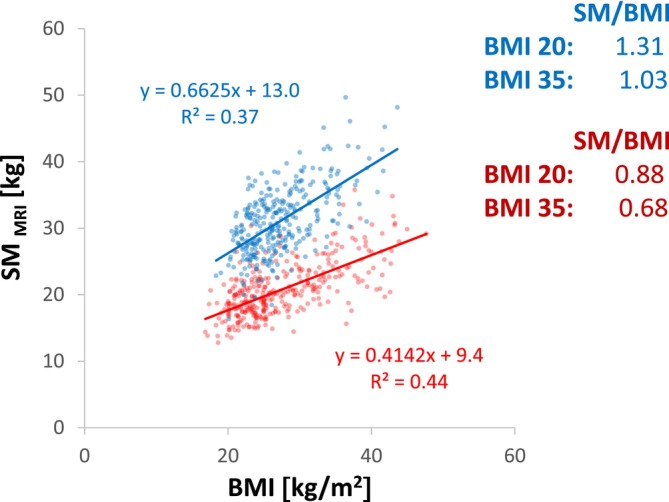
Relationships between whole body skeletal muscle mass (SM) assessed by magnetic resonance imaging and body mass index in 341 women (red symbols) and 319 men (blue symbols). Data are taken from Müller et al. (2018) [18]. Examples for a BMI of 20 and 35 kg/m^2^ show that SM cannot be normalized using SM/BMI because of a nonzero *y*‐intercept. In people with obesity, it is therefore normal to have a lower SM/BMI.

Alternative normalization approaches include the use of SM, ALST or FFM indexed to BMI, such as SM/BMI, ALST/BMI or FFM/BMI ([84]; for review, see [85]). However, ratio‐based indices assume a linear relationship and a zero intercept between the numerator and denominator—assumptions that are often violated in practice (see Figure 1).

Static normalization methods, such as FFM/BMI, adjust for BMI but do not account for variations in baseline body composition. In contrast, dynamic models incorporate baseline metrics and allow for more individualized interpretation.

For biologically meaningful interindividual comparisons, both FM and FFM (in kilograms) should be indexed to height squared (m^2^, FMI and FFMI), as both compartments scale with height with a power exponent of approximately two [86, 87]. Analogous to BMI, this approach provides size‐independent evaluation of SM as SMI and ALST as ALSTI.

### 1.5 Body Composition Method—Inherent Limitations Measuring Weight Changes

The *limited precision* of two‐compartment methods for assessing body composition constrains the interpretation of studies that report only small changes, since such differences may fall within the minimal detectable change of these techniques [88, 89, 90]. Consequently, conclusions suggesting that SGLT2 inhibitors that only lead to minor weight loss cause a disproportionate loss of FFM or SM [91] should be interpreted with caution.

#### 1.5.1 Regional Versus Whole‐Body SM Assessment During Weight Loss and Regain

Assessing SM at single anatomical sites, such as the lumbar vertebra (L3) or the thigh [78, 92], does not accurately reflect whole‐body SM changes during weight loss and subsequent regain. Although cross‐sectional imaging at L3 has been widely adopted as a reference standard for estimating total SM and adipose tissue, its sensitivity to detect longitudinal changes in SM remains limited [92]. In both sexes, SM recovery in the trunk appears to lag behind that of the extremities during weight regain [93], corroborating findings by Byrne et al. (2003) [94], who reported a preferential regain of lean soft tissue in the limbs compared to the trunk in weight‐regaining obese African American and White women.

#### 1.5.2 Altered Muscle Quality Limiting the Validity of Imaging Methods

In individuals with obesity, SM quality is often compromised due to alterations in fibre composition, myosteatosis and increased deposition of intramyocellular fat, intermuscular adipose tissue and collagen [95, 96, 97]. As a result, high SM mass may coexist with reduced muscle function, characterized by metabolic impairments (e.g., insulin resistance and mitochondrial dysfunction) and diminished physical performance [95, 98]. Notably, SM loss during weight reduction may be partially offset by improvements in muscle quality. Therefore, assessing functional outcomes, such as muscle strength, insulin sensitivity or biomarkers related to the muscle secretome [14], is critical for determining the clinical relevance of SM loss. MRI‐based body composition analysis from the UK Biobank utilizes advanced image processing to quantify fat‐free muscle volume (FFMV) in the anterior thighs using a dual‐echo Dixon VIBE protocol [78, 97]. FFMV is defined as the sum of all voxels with a fat fraction < 50%, thereby isolating metabolically active muscle tissue. Voxels corresponding to intermuscular adipose tissue—characterized by a higher fat content—are excluded by this threshold, making FFMV a robust and reproducible metric that avoids misclassification at muscle‐adipose boundaries.

#### 1.5.3 Altered FFM Composition Limiting the Validity of Two‐Compartment Body Composition Methods

Methodological issues also arise due to changes in the composition of FFM during weight loss that violate the underlying assumptions of two‐compartment methods. In the initial phase of weight loss (i.e., the first week), the ΔFFM/Δweight ratio is notably elevated, as weight loss predominantly consists of intracellular water bound to glycogen or protein or extracellular water associated with enhanced sodium excretion [45, 99, 100]. This instability gives rise to a violation of the method's underlying assumptions, such as the constant density and composition of FFM, and consequently hinders the accurate detection of initial changes in FM and FFM with two‐compartment body composition methods [88, 101].

The validity of two‐compartment methods is also affected by an increased hydration of FFM in people with obesity due to an elevated water fraction of FFM in connective tissue (i.e., adipose tissue). This is of particular relevance in the context of substantial weight loss, because even in the postobese state, the hydration of FFM remains higher as is evidenced by a higher extra‐to‐intracellular water ratio in weight‐reduced individuals (indicative of a persistently higher contribution of connective tissue to total lean mass) [102, 103]. Such an increase in hydration will thus lead to an overestimation of FFM after weight loss by BIA (owing to decreased resistance per body length) or deuterium dilution (because calculation of FFM from total body water would require a higher hydration factor) and a respective underestimation by densitometry or DXA (because of similar densities of fat and water; for a review, see [88]). Alternatively, a four‐compartment model that measures water and mineral constituents of FFM provides a more accurate measurement of FFM and FM independent of shifts in water balance with weight loss (see, e.g., [100, 104]).

#### 1.5.4 Altered FFM Composition Limiting the Utility of FFM as a Surrogate for SM

FFM is a heterogeneous compartment composed of SM, organ masses (e.g., brain, heart, liver and kidneys) and parts of connective tissue [90]. Obesity is characterized not only by excess adipose tissue but also by increases in the masses of organs and tissues of FFM (i.e., the ‘obesity tissue’): In individuals with a BMI > 29.9 in comparison to a BMI < 25, skin, liver and bone were identified as the primary contributors to the composition of ‘obesity tissue’ [105]. With respect to weight loss in patients with and without obesity, the major interest is on the loss in SM which may increase the risk of sarcopenia. Although SM is the primary component of total FFM, the use of FFM as a surrogate for SM is not without its inherent limitations. This is evident from the combined assessment of SM by whole body MRI and FFM by densitometry showing a decrease of SM as percentage of FFM from 44% (females) and 47% (males) in 18‐ to 29‐year‐old healthy adults to 39% in females and males at age > 60 years [106]. The decline in SM with age is potentially counterbalanced by an increase in connective tissue (i.e., an increase in the FFM component of adipose tissue), thereby rendering FFM insensitive to age‐related changes in SM.

Age‐ and weight‐related increases in connective tissue also influence ALST (defined as the sum of lean soft tissue mass in all four limbs as assessed by DXA [107]) which is widely used as a practical, cost‐effective surrogate for SM. However, the accuracy of ALST as a proxy for SM is affected by sex‐specific differences in fat distribution. DXA‐based comparisons of regional lean soft tissue with MRI‐derived SM show that ALST includes a lower proportion of SM in women, who tend to accumulate more FFM from adipose tissue in the limbs, compared to men, who store more adipose tissue in the trunk [108]. To account for these discrepancies, predictive equations estimating SM from ALST incorporate sex and age as covariates [109, 110]. Nonetheless, these variables contribute only modestly to improving prediction accuracy of cross‐sectional data.

**FIGURE 2:**
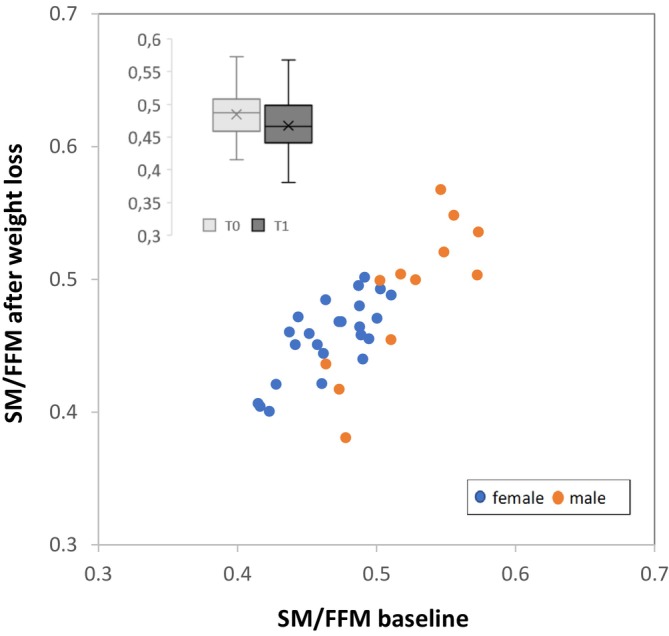
Ratio of skeletal muscle mass (SM, measured by whole body MRI) to fat‐free mass (FFM, measured by air displacement plethysmography) at baseline (T0) and after diet‐induced weight loss (T1; the ratio of SM to FFM decreased from 0.485 to 0.467; *p* < 0.001) in 36 men and women who lost ≥ 0.5 kg of FFM and SM. Data are taken from Pourhassan et al. (2013) [101].

During weight loss, the proportion of FFM attributable to SM undergoes significant changes. While SM constitutes approximately 45% of FFM in women and 49% in men [111], this proportion is not fixed and tends to decline with weight reduction. Data from Roux‐en‐Y gastric bypass (RYGB) patients demonstrate that although FFM accounted for only 16%–23% of total weight loss during the first postoperative year, SM represented a disproportionately large fraction (92%–97%) of the FFM lost [112]. Comparable findings were observed in a cohort of 36 healthy adults (12 males, 24 females) who experienced a mean diet‐induced weight loss of −10.8 ± 4.9 kg. In this group, FFM decreased by −2.33 ± 1.98 kg (assessed by ADP), while SM declined by −2.26 ± 1.66 kg (assessed by whole‐body MRI, data are taken from [101]). Although FFM accounted for 22% of total weight loss, SM contributed to 97% of the FFM reduction, and the contribution of SM to FFM decreased from 48.5% to 46.7% (*p* < 0.001, Figure 2).

This pattern reflects a preferential loss of SM over other lean compartments, particularly connective tissue, which is largely preserved during weight loss. Consequently, weight loss alters the composition of lean mass, leading to an increased ratio of extracellular to intracellular water that is a marker of higher connective tissue content and reduced muscle mass [102]. These compositional changes persist postweight loss and highlight a key limitation of using FFM as a proxy for SM in clinical weight loss trials. Importantly, since adipose tissue contains a notable lean component of up to 15% FFM [113], it had been suggested that adipose tissue loss could contribute substantially to FFM reduction. However, adipose tissue shrinks primarily through lipid depletion within adipocytes rather than via degradation of the tissue matrix [114]. Therefore, the lean fraction of adipose tissue, including extracellular water, remains relatively stable, and most of the observed FFM loss is actually attributable to SM and intracellular water ([102, 112], Figure 2).

These findings emphasize that a ‘normal’ BMI is not an appropriate treatment goal in weight‐reduced individuals, as it may conceal significant muscle loss. Accurate assessment of SM—rather than total FFM—is essential for evaluating the quality of weight loss and for preserving musculoskeletal health during obesity treatment.

### 1.6 What Is a Healthy Target for Weight Loss?

Because weight reduction induces a sustained alteration in FFM that is characterized by a disproportionately greater loss of SM compared with connective tissue ([102, 112]; own results, see above), individuals who have experienced substantial weight loss require a higher FFM to preserve the same amount of SM as those with an equivalent BMI but without prior weight loss. This consideration is highly relevant in the context of AOMs, which can produce profound reductions in body weight, with a notable proportion of patients achieving normal weight. In the SELECT trial (Semaglutide 2.4 mg, adults without diabetes), 12.0% of treated participants reached a BMI < 25 kg/m^2^ at 104 weeks compared with 1.2% in the placebo group [115]. Similarly, in a post hoc analysis of SURMOUNT‐1 (Tirzepatide, adults without diabetes), approximately 18% of participants achieved a BMI < 25 kg/m^2^ by week 176 [116]. Even more pronounced results were reported with Retatrutide, a GIP–GLP‐1–glucagon receptor triple agonist: Participants achieved a mean weight reduction of 24.2% after 48 weeks, with weight loss trajectories showing no plateau by the end of treatment [117]. At the highest dose (12 mg), 26% of patients achieved weight loss exceeding 30%. Notably, 14 participants reached a BMI ≤ 22 kg/m^2^ (1 in the placebo group and 13 in the Retatrutide groups), of whom 8 required protocol‐mandated dose reductions due to excessively low BMI [117].

#### 1.6.1 Is the Extent of Weight Loss an Appropriate Criterion for Treatment Success?

We therefore aimed to establish evidence‐based criteria for defining a maximal weight loss target. Baseline SMI was estimated from FFM, height, weight, sex and age. SMI after weight loss was then calculated under the assumption that the entire reduction in FFM reflects loss of SM ([112]; own results, see above). This postloss SMI was compared with the expected normal SMI for the individual, derived from BMI, sex and age, using percentile thresholds based on the SMI‐SDS.

1 Calculation of baseline SMI based on FFMI and demographic data

A cross‐sectional dataset of 280 healthy adults (158 females, 122 males; age 41 ± 14 years; BMI 28 ± 6 kg/m^2^; data were taken from our database published in [18]) was analysed. SM was measured by whole‐body MRI and FFM by densitometry. SM was 45% and 49% of FFM in women and men (*p* < 0.001). In a stepwise multiple regression, 87% of the variance in SM index (SMI, kg/m^2^) was explained:

$$\begin{array}{c} \mathrm{SMI}=0.431\times \text{FFMI}+0.019\times \text{weight}+0.630\times \mathrm{sex}\\ -0.011\times \mathrm{age}-0.736\;\left(\text{female}=0,\text{male}=1;\mathrm{SEE}=0.635\right). \end{array}$$

This equation allows the calculation of baseline SMI for an individual patient.

2 SMI after weight loss can then be calculated from the decrease in FFM with weight loss, assuming ΔFFM = ΔSM.
3 SMI after weight loss can be compared with a normal SMI for a given BMI, sex and age using the equations by Enderle et al. (2023) [81] (Table 1). An SMI‐standard deviation score (SMI‐SDS) is calculated as follows: SMI‐SDS = (SMI observed—SMI predicted) / SEE. An SMI‐SDS of −0.67 corresponds to the 25th percentile of the reference population and may therefore define the maximal safe weight loss target.

**FIGURE 3:**
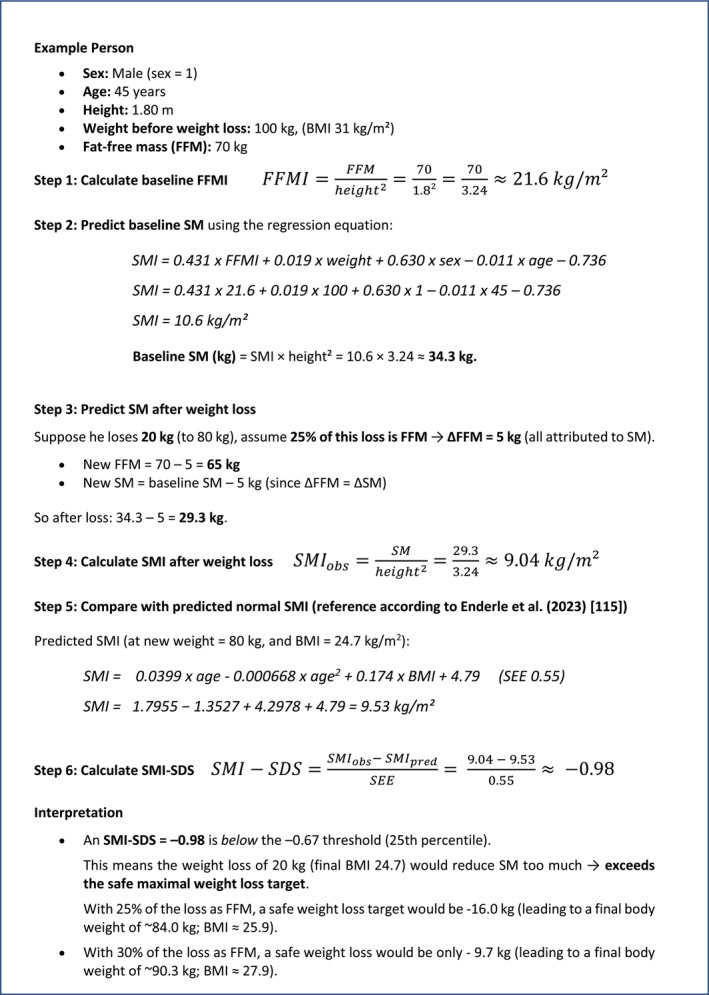
Estimation of the maximum safe weight loss based on preservation of skeletal muscle mass. An example is shown for a 45‐year‐old man (height 1.80 m, baseline body weight 100 kg, BMI 31 kg/m^2^, FFM 70 kg). Baseline skeletal muscle mass (SM) was estimated from fat‐free mass index (FFMI). After simulated weight loss, skeletal muscle mass was assumed to decline in proportion to the loss of FFM. The resulting skeletal muscle index (SMI) was compared with the age‐ and BMI‐specific reference values by Enderle et al. to calculate the SMI standard deviation score (SMI‐SDS). An SMI‐SDS below −0.67 (25th percentile) was considered indicative of excessive skeletal muscle loss. In this example, assuming that 25% of weight loss consists of FFM, the maximum safe weight loss is approximately 16.0 kg (final body weight = 84.0 kg; BMI = 25.9 kg/m^2^). If 30% of the weight loss is attributable to FFM, the maximum safe weight loss decreases to approximately 9.7 kg (final body weight = 90.3 kg; BMI = 27.9 kg/m^2^). The example illustrates how the proportion of FFM loss substantially influences the recommended upper limit of weight reduction.

Figure 3 gives an example how to apply this method using data from a hypothetical patient. This example also shows that an a priori estimation of the proportion of weight loss attributable to FFM is essential for defining a safe upper limit of weight reduction. This estimation can be significantly enhanced by using the NIH body weight planner, which incorporates key individual parameters—including baseline body weight and composition, sex, age, height, physical activity level and observed postintervention weight (‘goal weight’)—along with the duration of the intervention (https://www.niddk.nih.gov/health‐information/weight‐management/body‐weight‐planner). Beyond planning, this tool is also valuable for monitoring treatment trajectories, helping to detect excessively rapid weight loss that may require dose adjustment or modification of AOM to avoid disproportionate FFM depletion.

A review of 28 clinical trials involving GLP‐1 receptor agonists revealed high variability in the proportion of FFM loss relative to total weight loss (20%–40%), with > 25% FFM loss reported in most studies [118]. In the SURMOUNT‐1 trial, treatment with the dual GLP‐1/GIP receptor agonist Tirzepatide resulted in a 33.9% reduction in FM and a 10.9% reduction in FFM, indicating that FFM accounted for approximately 33% of total weight loss [36]. Similarly, data from the Retatrutide trial showed that at the highest dose (12 mg), individuals with T2D experienced an 18.7% reduction in FM and a 10.5% reduction in FFM, with FFM representing approximately 38% of total weight reduction [37]. The mean proportion of FFM lost in these trials clearly shows the risk of setting overly ambitious weight loss goals. This is also supported by Figure S4 that shows, with the exception of two studies, the result of trials involving incretin receptor agonist therapy lies substantially above the modified Forbes curve [49], indicating a disproportionately high loss of FFM. In contrast, the positioning of lifestyle intervention studies largely depends on whether an exercise component was included. When exercise was part of the intervention, changes in body composition were consistent with—or even more favourable than—those predicted by the model.

Because discontinuation of AOMs typically leads to weight regain—driven primarily by increases in FM, while FFM is only partially restored (reviewed in [119])—treatment strategies should prioritize the preservation of SM. Achieving this requires a multimodal approach that combines a high‐protein diet, resistance exercise and, where appropriate, pharmacological interventions aimed at minimizing SM loss [61, 120]. The effectiveness of such integrated strategies should be systematically evaluated to strengthen the evidence base for this recommendation.

### 1.7 Summary and Conclusion

This review highlights two complementary strategies for evaluating weight loss: a dynamic model that predicts expected changes in FFM based on physiological determinants and a static approach that benchmarks body composition before and after weight loss against reference populations. By integrating these approaches with a regression‐based prediction of normal SMI, we define evidence‐based criteria for a maximal safe weight loss target. An SMI‐SDS below −0.67 (25th percentile) may serve as a threshold indicating disproportionate muscle loss.

Linking clinical data to reference thresholds for SM provides a practical framework for setting individualized, body composition‐based treatment goals in the era of potent AOMs. Safe and effective obesity care requires moving beyond weight or BMI alone and incorporating nutritional therapy, structured physical training and AOM to achieve durable and healthy outcomes.

## Conflicts of Interest

The authors declare no conflicts of interest.

## Supporting information


**Figure S4:** Conceptual framework for assessing changes in body composition during weight loss, illustrated with original data from selected studies. An energy deficit—induced by lifestyle changes or anti‐obesity pharmacotherapy—leads to weight loss that is partitioned between fat mass (FM) and fat‐free mass (FFM). The relative contribution of each compartment is determined by baseline adiposity (black Forbes curve) and further modulated by factors such as protein intake, energy deficit per day and resistance exercise. Each data point represents an individual study or study arm in which FM loss exceeded 5 kg and was assessed using dual‐energy X‐ray absorptiometry (DXA) or a four‐compartment (4C) model [101, 121–136].
