# Supplementary material for: Defining Healthy Weight Loss and Target Weight in the Era of Highly Effective Treatment of Patients With Obesity
Source: J Cachexia Sarcopenia Muscle. 2026 Jul 15;17(4):e70334. doi: 10.1002/jcsm.70334 (PMC13371635; doi:10.1002/jcsm.70334)
Supplement: Supplementary file 1 — Figure S4: Conceptual framework for assessing changes in body composition during weight loss, illustrated with original data from selected studies. An energy deficit—induced by lifestyle changes or anti‐obesity pharmacotherapy—leads to weight loss that is partitioned between fat mass (FM) and fat‐free mass (FFM). The relative contribution of each compartment is determined by baseline adiposity (black Forbes curve) and further modulated by factors such as protein intake, energy deficit per day and resistance exercise. Each data point represents an individual study or study arm in which FM loss exceeded 5 kg and was assessed using dual‐energy X‐ray absorptiometry (DXA) or a four‐compartment (4C) model [101, 121–136]. [file JCSM-17-e70334-s001.pptx]

## Slide 1
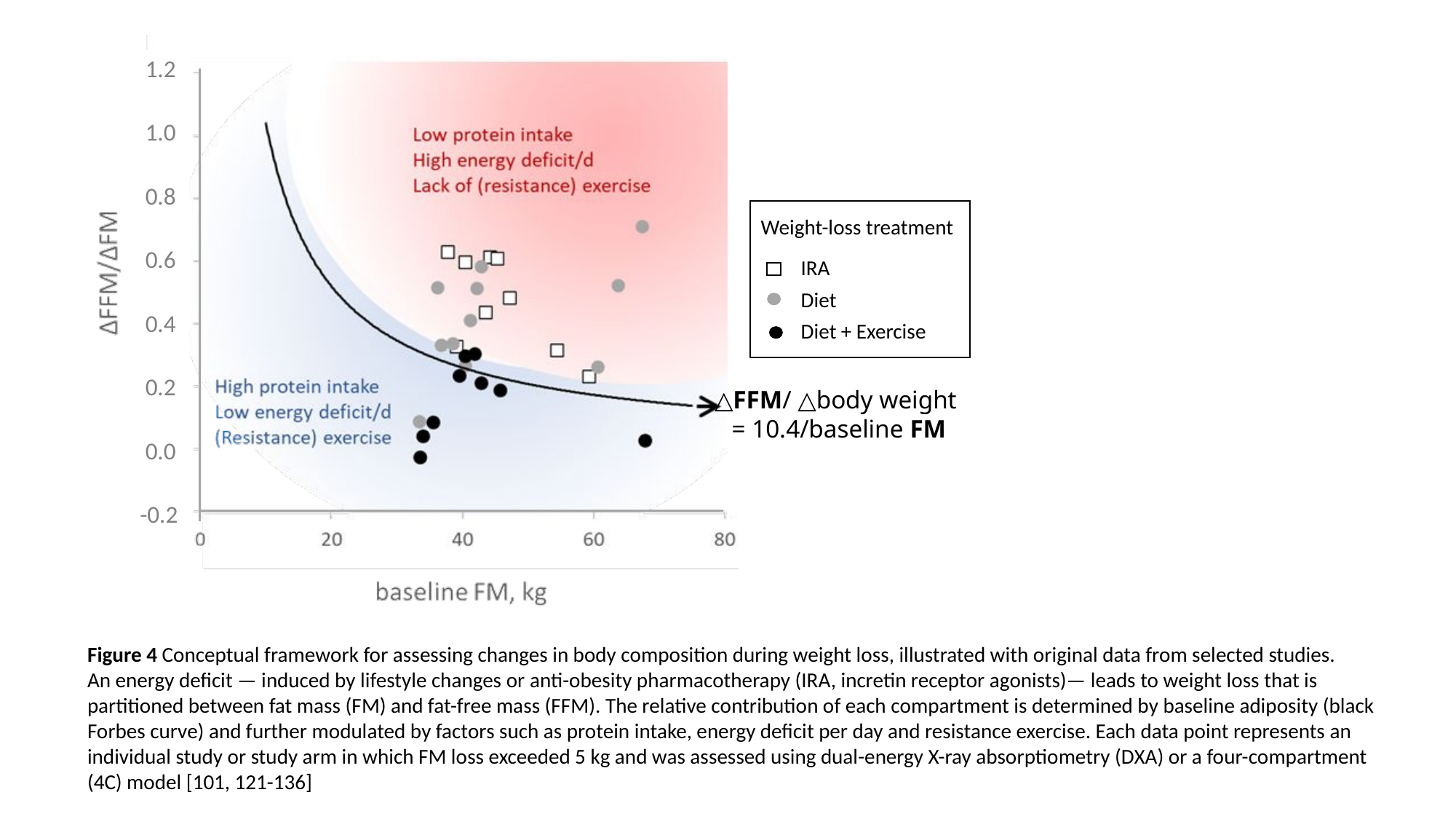

1.2
 1.0
 0.8
 0.6
 0.4
 0.2
 0.0
-0.2
Weight-loss treatment
IRA
Diet
Diet + Exercise
△FFM/ △body weight
= 10.4/baseline FM
Figure 4 Conceptual framework for assessing changes in body composition during weight loss, illustrated with original data from selected studies.
An energy deficit — induced by lifestyle changes or anti-obesity pharmacotherapy (IRA, incretin receptor agonists)— leads to weight loss that is partitioned between fat mass (FM) and fat-free mass (FFM). The relative contribution of each compartment is determined by baseline adiposity (black Forbes curve) and further modulated by factors such as protein intake, energy deficit per day and resistance exercise. Each data point represents an individual study or study arm in which FM loss exceeded 5 kg and was assessed using dual-energy X-ray absorptiometry (DXA) or a four-compartment (4C) model [101, 121-136]

## Slide 2
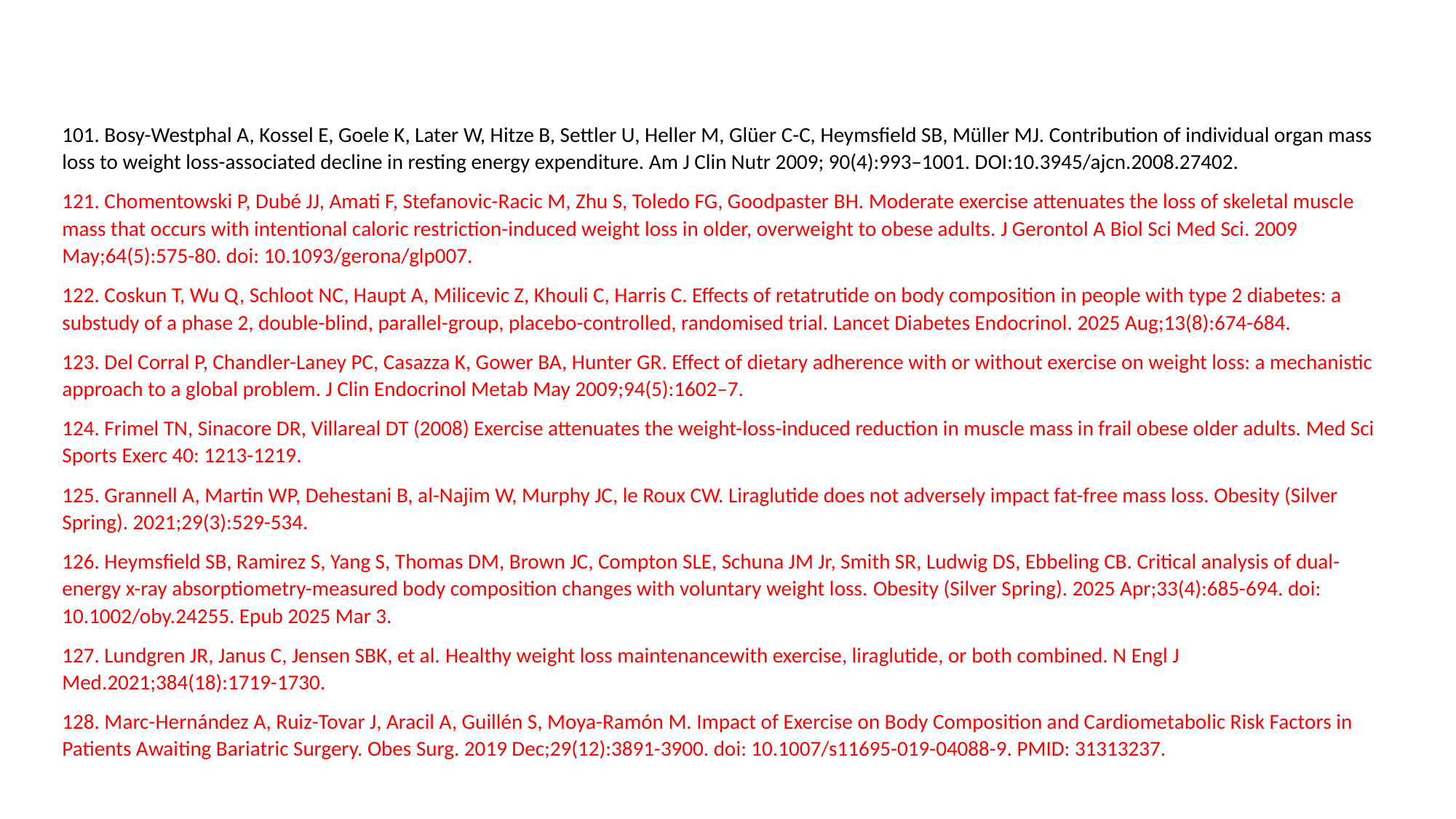

101. Bosy-Westphal A, Kossel E, Goele K, Later W, Hitze B, Settler U, Heller M, Glüer C-C, Heymsfield SB, Müller MJ. Contribution of individual organ mass loss to weight loss-associated decline in resting energy expenditure. Am J Clin Nutr 2009; 90(4):993–1001. DOI:10.3945/ajcn.2008.27402.
121. Chomentowski P, Dubé JJ, Amati F, Stefanovic-Racic M, Zhu S, Toledo FG, Goodpaster BH. Moderate exercise attenuates the loss of skeletal muscle mass that occurs with intentional caloric restriction-induced weight loss in older, overweight to obese adults. J Gerontol A Biol Sci Med Sci. 2009 May;64(5):575-80. doi: 10.1093/gerona/glp007.
122. Coskun T, Wu Q, Schloot NC, Haupt A, Milicevic Z, Khouli C, Harris C. Effects of retatrutide on body composition in people with type 2 diabetes: a substudy of a phase 2, double-blind, parallel-group, placebo-controlled, randomised trial. Lancet Diabetes Endocrinol. 2025 Aug;13(8):674-684.
123. Del Corral P, Chandler-Laney PC, Casazza K, Gower BA, Hunter GR. Effect of dietary adherence with or without exercise on weight loss: a mechanistic approach to a global problem. J Clin Endocrinol Metab May 2009;94(5):1602–7.
124. Frimel TN, Sinacore DR, Villareal DT (2008) Exercise attenuates the weight-loss-induced reduction in muscle mass in frail obese older adults. Med Sci Sports Exerc 40: 1213-1219.
125. Grannell A, Martin WP, Dehestani B, al-Najim W, Murphy JC, le Roux CW. Liraglutide does not adversely impact fat-free mass loss. Obesity (Silver Spring). 2021;29(3):529-534.
126. Heymsfield SB, Ramirez S, Yang S, Thomas DM, Brown JC, Compton SLE, Schuna JM Jr, Smith SR, Ludwig DS, Ebbeling CB. Critical analysis of dual-energy x-ray absorptiometry-measured body composition changes with voluntary weight loss. Obesity (Silver Spring). 2025 Apr;33(4):685-694. doi: 10.1002/oby.24255. Epub 2025 Mar 3.
127. Lundgren JR, Janus C, Jensen SBK, et al. Healthy weight loss maintenancewith exercise, liraglutide, or both combined. N Engl J Med.2021;384(18):1719-1730.
128. Marc-Hernández A, Ruiz-Tovar J, Aracil A, Guillén S, Moya-Ramón M. Impact of Exercise on Body Composition and Cardiometabolic Risk Factors in Patients Awaiting Bariatric Surgery. Obes Surg. 2019 Dec;29(12):3891-3900. doi: 10.1007/s11695-019-04088-9. PMID: 31313237.

## Slide 3
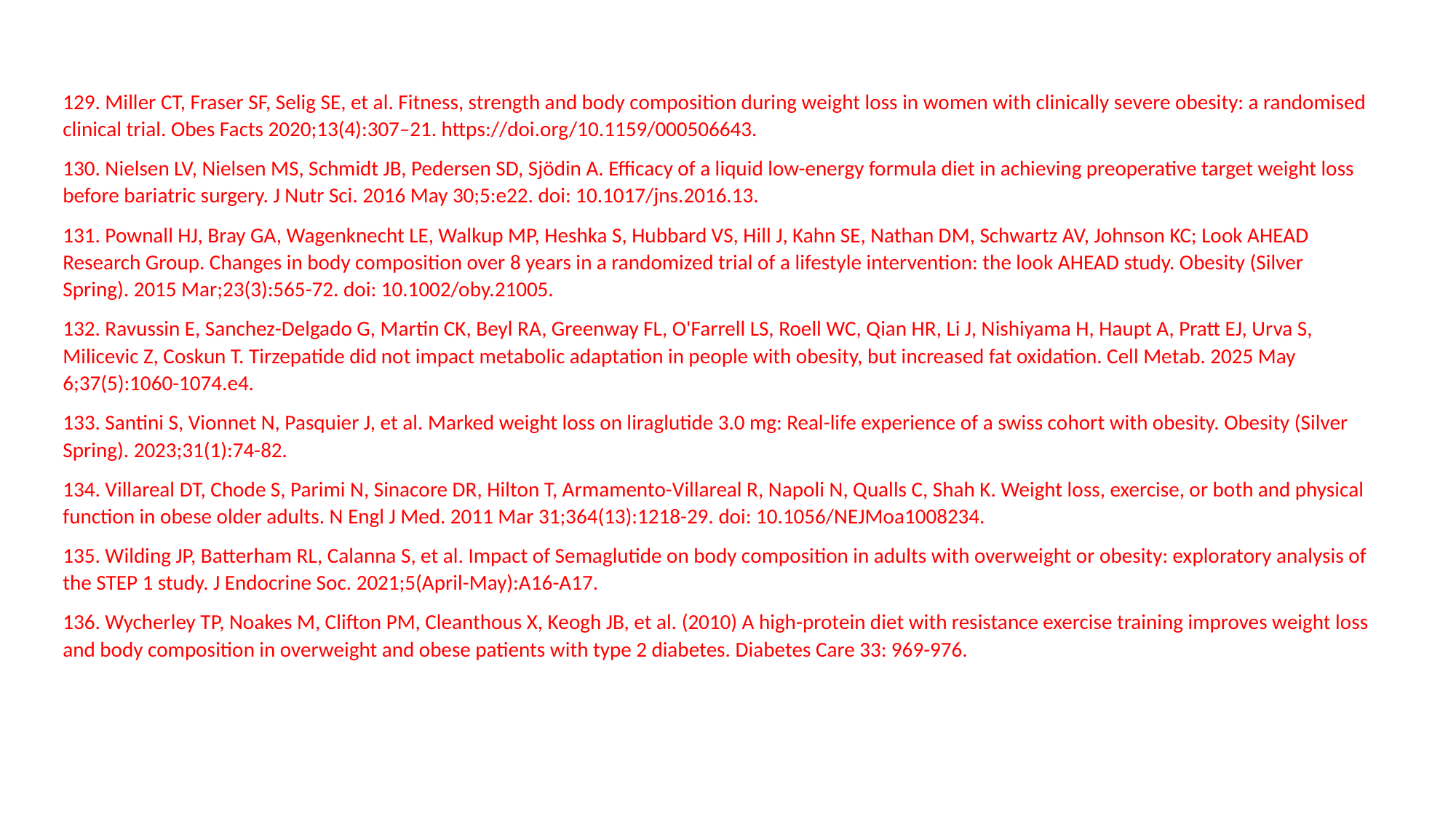

129. Miller CT, Fraser SF, Selig SE, et al. Fitness, strength and body composition during weight loss in women with clinically severe obesity: a randomised clinical trial. Obes Facts 2020;13(4):307–21. https://doi.org/10.1159/000506643.
130. Nielsen LV, Nielsen MS, Schmidt JB, Pedersen SD, Sjödin A. Efficacy of a liquid low-energy formula diet in achieving preoperative target weight loss before bariatric surgery. J Nutr Sci. 2016 May 30;5:e22. doi: 10.1017/jns.2016.13.
131. Pownall HJ, Bray GA, Wagenknecht LE, Walkup MP, Heshka S, Hubbard VS, Hill J, Kahn SE, Nathan DM, Schwartz AV, Johnson KC; Look AHEAD Research Group. Changes in body composition over 8 years in a randomized trial of a lifestyle intervention: the look AHEAD study. Obesity (Silver Spring). 2015 Mar;23(3):565-72. doi: 10.1002/oby.21005.
132. Ravussin E, Sanchez-Delgado G, Martin CK, Beyl RA, Greenway FL, O'Farrell LS, Roell WC, Qian HR, Li J, Nishiyama H, Haupt A, Pratt EJ, Urva S, Milicevic Z, Coskun T. Tirzepatide did not impact metabolic adaptation in people with obesity, but increased fat oxidation. Cell Metab. 2025 May 6;37(5):1060-1074.e4.
133. Santini S, Vionnet N, Pasquier J, et al. Marked weight loss on liraglutide 3.0 mg: Real-life experience of a swiss cohort with obesity. Obesity (Silver Spring). 2023;31(1):74-82.
134. Villareal DT, Chode S, Parimi N, Sinacore DR, Hilton T, Armamento-Villareal R, Napoli N, Qualls C, Shah K. Weight loss, exercise, or both and physical function in obese older adults. N Engl J Med. 2011 Mar 31;364(13):1218-29. doi: 10.1056/NEJMoa1008234.
135. Wilding JP, Batterham RL, Calanna S, et al. Impact of Semaglutide on body composition in adults with overweight or obesity: exploratory analysis of the STEP 1 study. J Endocrine Soc. 2021;5(April-May):A16-A17.
136. Wycherley TP, Noakes M, Clifton PM, Cleanthous X, Keogh JB, et al. (2010) A high-protein diet with resistance exercise training improves weight loss and body composition in overweight and obese patients with type 2 diabetes. Diabetes Care 33: 969-976.

## Slide 4
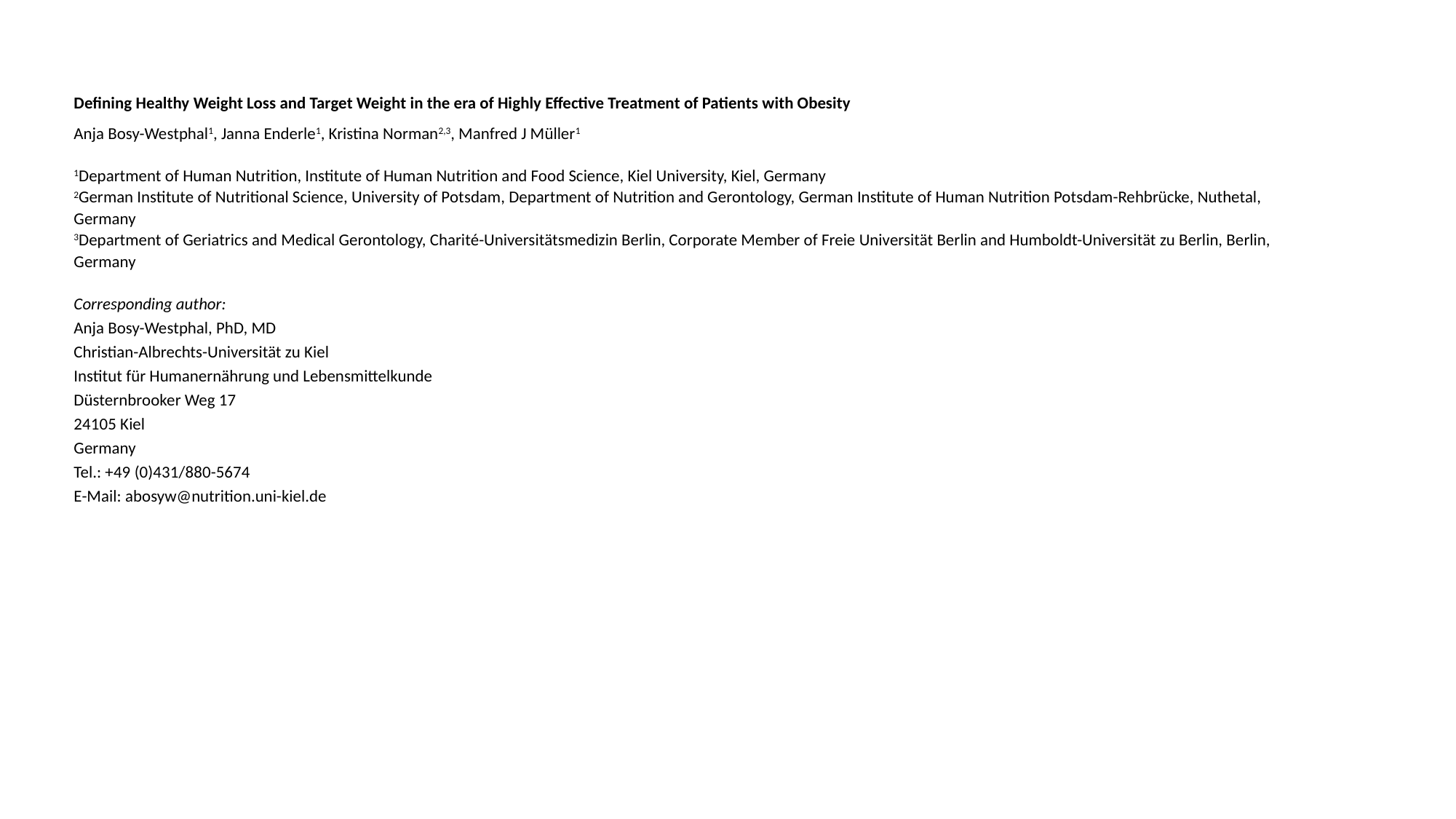

Defining Healthy Weight Loss and Target Weight in the era of Highly Effective Treatment of Patients with Obesity
Anja Bosy-Westphal1, Janna Enderle1, Kristina Norman2,3, Manfred J Müller1
1Department of Human Nutrition, Institute of Human Nutrition and Food Science, Kiel University, Kiel, Germany
2German Institute of Nutritional Science, University of Potsdam, Department of Nutrition and Gerontology, German Institute of Human Nutrition Potsdam-Rehbrücke, Nuthetal, Germany
3Department of Geriatrics and Medical Gerontology, Charité-Universitätsmedizin Berlin, Corporate Member of Freie Universität Berlin and Humboldt-Universität zu Berlin, Berlin, Germany
Corresponding author:
Anja Bosy-Westphal, PhD, MD
Christian-Albrechts-Universität zu Kiel
Institut für Humanernährung und Lebensmittelkunde
Düsternbrooker Weg 17
24105 Kiel
Germany
Tel.: +49 (0)431/880-5674
E-Mail: abosyw@nutrition.uni-kiel.de
